# Immune system dysregulation in the pathogenesis of non-alcoholic steatohepatitis: unveiling the critical role of T and B lymphocytes

**DOI:** 10.3389/fimmu.2024.1445634

**Published:** 2024-08-01

**Authors:** Merve Cebi, Yusuf Yilmaz

**Affiliations:** ^1^ Department of Medical Biology, School of Medicine, Recep Tayyip Erdoğan University, Rize, Türkiye; ^2^ Department of Gastroenterology, School of Medicine, Recep Tayyip Erdoğan University, Rize, Türkiye; ^3^ The Global NASH Council, Washington, DC, United States

**Keywords:** non-alcoholic fatty liver disease, non-alcoholic steatohepatitis, T lymphocytes, B lymphocytes, fibrosis, inflammation, pathogenesis

## Abstract

Non-alcoholic fatty liver disease (NAFLD), characterized by the excessive accumulation of fat within the cytoplasm of hepatocytes (exceeding 5% of liver weight) in individuals without significant alcohol consumption, has rapidly evolved into a pressing global health issue, affecting approximately 25% of the world population. This condition, closely associated with obesity, type 2 diabetes, and the metabolic syndrome, encompasses a spectrum of liver disorders ranging from simple steatosis without inflammation to non-alcoholic steatohepatitis (NASH) and cirrhotic liver disease. Recent research has illuminated the complex interplay between metabolic and immune responses in the pathogenesis of NASH, underscoring the critical role played by T and B lymphocytes. These immune cells not only contribute to necroinflammatory changes in hepatic lobules but may also drive the onset and progression of liver fibrosis. This narrative review aims to provide a comprehensive exploration of the effector mechanisms employed by T cells, B cells, and their respective subpopulations in the pathogenesis of NASH. Understanding the immunological complexity of NASH holds profound implications for the development of targeted immunotherapeutic strategies to combat this increasingly prevalent and burdensome metabolic liver disease.

## 1 Introduction

Non-alcoholic fatty liver disease (NAFLD) is a chronic liver disorder characterized by excessive hepatic lipid accumulation, exceeding 5% of its weight, in individuals without significant alcohol consumption (1, 2). Closely linked to metabolic risk factors such as obesity, type 2 diabetes, and dyslipidemia, NAFLD is commonly conceptualized as the hepatic manifestation of the metabolic syndrome (3). The global prevalence of NAFLD has witnessed a dramatic increase over the past 30 years, with estimates surging from 25% to an alarming 38% (4, 5). This upward trajectory shows no signs of abating, as projections indicate that, by the year 2040, approximately 55% of the world’s population will be affected by NAFLD (6).

**Figure 1:**
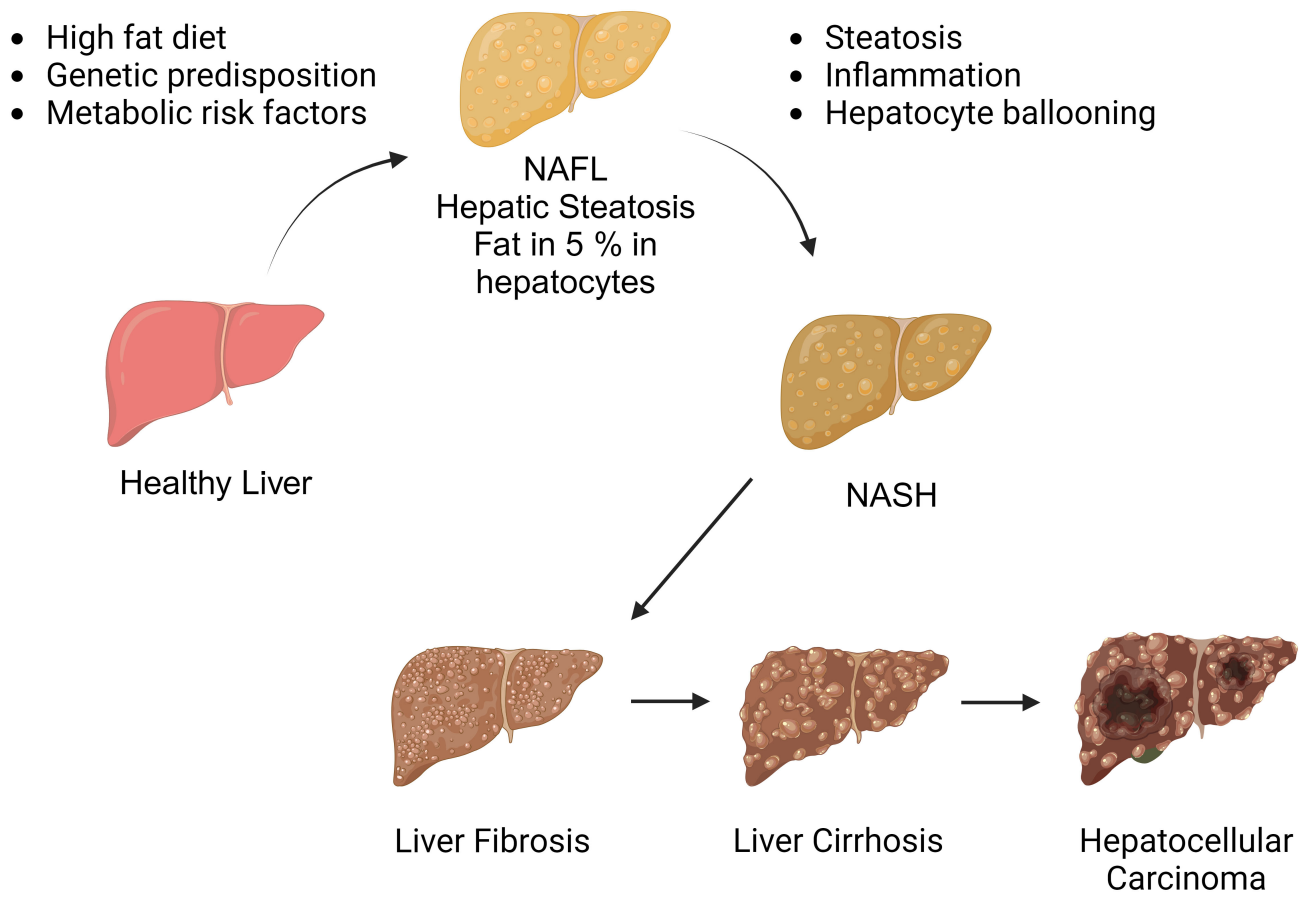
The spectrum of non-alcoholic fatty liver disease. Non-alcoholic fatty liver disease encompasses a spectrum of liver conditions, ranging from simple steatosis to nonalcoholic steatohepatitis (NASH). NASH is characterized by liver inflammation and hepatocellular injury, which can progress to cirrhotic liver disease. In some cases, cirrhosis may be further complicated by hepatocellular carcinoma. (Created with BioRender.com).

The NAFLD spectrum (**Figure 1**) encompasses a series of progressive stages, commencing with simple steatosis, characterized by liver fat accumulation without inflammation. As the condition advances, it may progress to non-alcoholic steatohepatitis (NASH), marked by necroinflammatory changes in hepatic lobules. Notably, NASH can lead to the development of liver fibrosis, which is the primary adverse prognostic determinant. In the most severe cases, NAFLD may ultimately culminate in serious complications such as cirrhotic liver disease and hepatocellular carcinoma (HCC) (7, 8). The current treatment options for NASH are notably limited, with weight loss and lifestyle modifications being the primary recommendations (9). However, necroinflammation is recognized as the main driving factor behind the progression from simple steatosis to NASH. Therefore, understanding the pathophysiological mechanisms that trigger liver inflammation in NASH and how inflammatory changes may progress to fibrotic responses and hepatic carcinogenesis.

In the context of liver damage, the innate immune response is initially prominent, whereas adaptive immunity becomes more effective in sustaining chronic hepatic inflammation. Notably, chronic hepatic inflammation is the primary driver in the progression of NASH to liver fibrosis and/or cirrhosis (10). Additionally, oxidative stress – a common feature in NASH pathogenesis – triggers the release of chemokines and pro-inflammatory cytokines from hepatocytes and liver endothelial cells, playing a crucial role in lymphocyte migration to the hepatic parenchyma. T and B lymphocytes that migrate to the liver interact with oxidative stress-derived antigens and play an active role in liver damage through the secretion of pro-inflammatory molecules (10).

Building on these premises, this review explores the migration of adaptive immune cells to the liver, the activation and functional properties of T and B cells, and their potential roles in the pathogenesis of inflammation and fibrosis. In addition, potential therapeutic strategies to regulate adaptive immune responses are examined.

## 2 Inflammation and leukocyte migration in NASH

In response to tissue injury or infection, an inflammatory response is initiated, resulting in the secretion of a diverse array of inflammatory mediators, including cytokines and chemokines. These molecules serve to activate cellular defense mechanisms and facilitate tissue repair processes. However, chronic inflammation can lead to persistent pathological changes, exacerbating tissue damage and potentially contributing to the progression of necroinflammation and fibrosis in patients with NAFLD. In general, the inflammatory response in the liver can be triggered by both extrahepatic sources, such as adipose tissue and the intestine, as well as intrahepatic factors, including lipotoxicity, innate immune responses, and cell death pathways.

**Figure 2:**
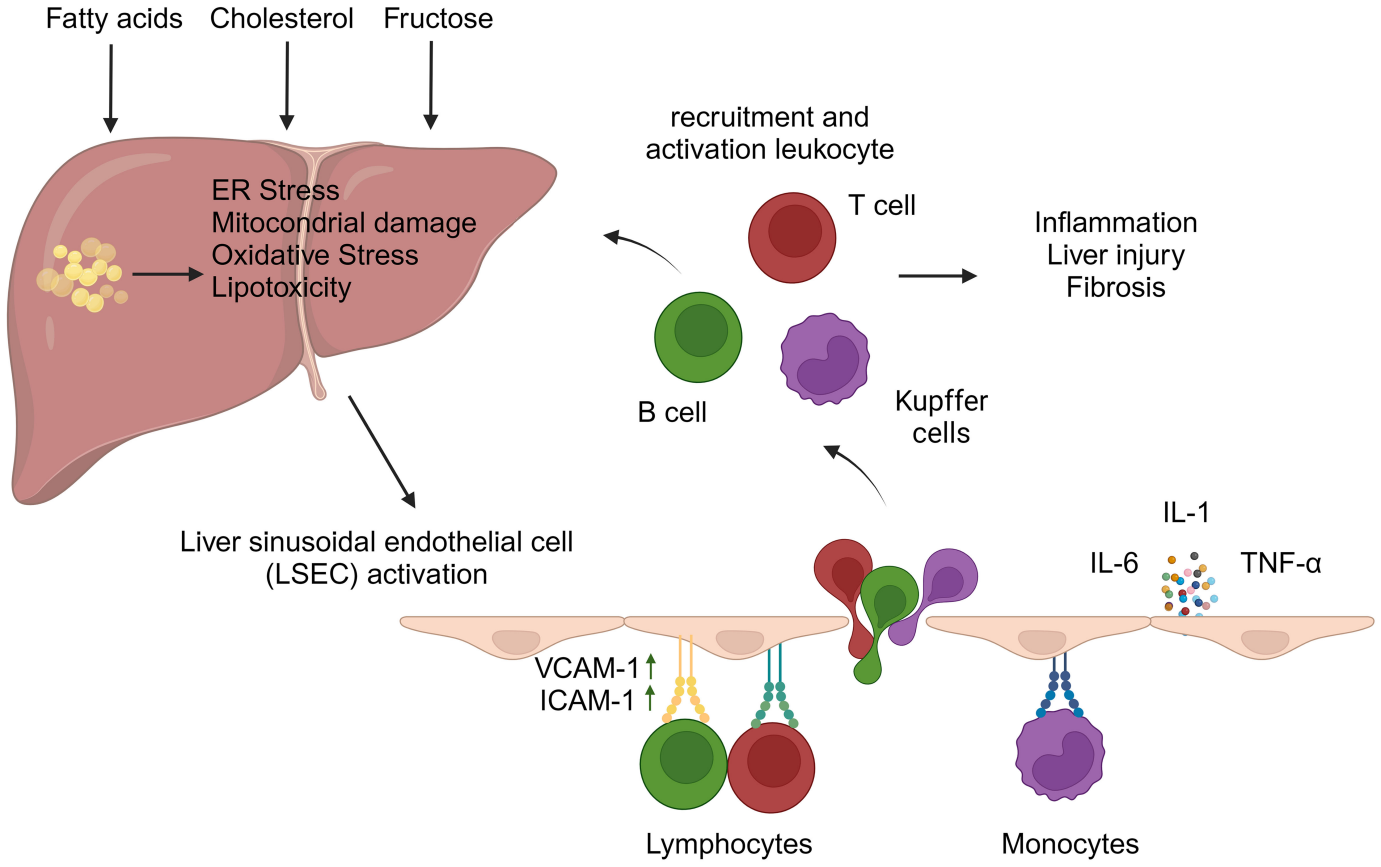
Liver sinusoidal endothelial cell activation and leukocyte migration in hepatic inflammation. The accumulation of fatty acids, fructose, and/or cholesterol within hepatocytes can trigger cellular stresses, including oxidative stress, mitochondrial damage, and endoplasmic reticulum (ER) stress. These events contribute to the development of liver inflammation. In this inflammatory microenvironment, liver sinusoidal endothelial cells (LSECs) become activated and express adhesion molecules, particularly vascular cell adhesion molecule-1 (VCAM-1) and intercellular adhesion molecule-1 (ICAM-1), which facilitate leukocyte migration. Furthermore, activated LSECs secrete pro-inflammatory cytokines, such as tumor necrosis factor-alpha, interleukin-6, and interleukin-1. These molecules exacerbate liver inflammation and promote the adhesion of leukocytes to VCAM-1 and ICAM-1 expressed on the LSEC surface. The migration of leukocytes into the liver contributes to both intrahepatic inflammation and fibrosis. (Created with BioRender.com).

The pathogenesis of NASH is associated with a variety of cellular stresses in hepatocytes, including endoplasmic reticulum (ER) stress, mitochondrial dysfunction, oxidative stress, and lipotoxicity (11). These processes can be attributed to several factors, such as the accumulation of saturated fatty acids, increased *de novo* lipogenesis resulting from elevated fructose consumption, or the accumulation of cholesterol in the ER. Notably, fructose has been shown to induce intestinal hyperpermeability, which may lead to the activation of hepatic immune cells through gut-derived inflammatory signals (12). This cascade of events initiates liver inflammation and ultimately results in hepatocyte death. Cellular stress within hepatocytes can also lead to the release of pro-inflammatory mediators, which subsequently amplify the inflammatory response and contribute to the progression of fibrosis. The pro-inflammatory milieu in the liver triggers the migration of immune cells from the circulation into the liver parenchyma. This process is typically orchestrated by liver sinusoidal endothelial cells (LSECs), a prominent non-parenchymal cell population in the liver. Due to their unique location between the blood and liver parenchyma, LSECs play a crucial role in regulating liver cell homeostasis (13). Triglyceride accumulation in hepatocytes can lead to cellular damage, which may result in the activation of these cells and the acquisition of pro-inflammatory properties – a process that contributes to the development of NASH (14). Under physiological conditions, LSECs play a crucial role in mitigating excessive inflammatory responses in the liver by removing oxidized low-density lipoprotein (LDL) cholesterol and advanced glycation end-products from the circulation (15). Additionally, LSECs are capable of presenting antigens to naive CD4+ and CD8+ T cells, typically promoting an anti-inflammatory phenotype. *In vitro* experiments have demonstrated that when LSECs are cultured with naive CD4+ T cells, there is a notable increase in interleukin (IL)-10 expression (16). Similarly, interactions between LSECs and naive CD8+ T cells result in an elevated expression of the co-inhibitory molecule B7-H1, rather than the co-stimulatory molecules CD80/CD86, on CD8+ T cells (17). Moreover, LSECs facilitate the induction of FoxP3+ regulatory T cells (Tregs) in a transforming growth factor (TGF)-β-dependent manner (18) and play a crucial role in regulating the migration of immune cells to the liver. Under physiological conditions, LSECs exhibit minimal expression of selectins and adhesion molecules. However, when activated by the liver inflammatory environment, they upregulate classical adhesion molecules – including intercellular adhesion molecule-1 (ICAM-1) and vascular cell adhesion molecule-1 (VCAM-1) – which are essential for leukocyte migration (**Figure 2**) (19). As NAFLD progresses, studies utilizing mouse models of NASH have demonstrated that LSECs promote the expression of VCAM-1 (20, 21). In the inflammatory milieu, activated LSECs also secrete pro-inflammatory mediators such as tumor necrosis factor (TNF)-α, IL-1, and IL-6, further exacerbating liver inflammation (22). In addition, inhibition of VCAM-1 has been shown to reduce leukocyte migration to the liver and mitigate the development of NASH in a diet-induced NASH mouse model (20, 23).

Lymphocyte infiltration in the liver is a pivotal factor in the pathogenesis of NASH. This phenomenon, documented in both human studies and rodent models, is significantly driven by circulating antigens resulting from oxidative stress (10). The regulatory capacity of LSECs over lymphocyte behavior is critically important in this context. Although the liver is generally characterized by immunological tolerance, the progression of NASH is associated with increased intrahepatic inflammation, which subsequently alters the immunological profile of LSECs. This alteration fosters an environment conducive to lymphocyte activation, thereby exacerbating hepatic injury.

## 3 T cells in NASH

T cells, which are essential components of the adaptive immune system, play a crucial role in the pathogenesis of NASH. Notably, they modulate immune responses by regulating the function of other immune cells. The primary subsets of T cells include CD4+ helper T (Th) cells and CD8+ cytotoxic T cells. CD4+ Th cells are further categorized into Th1, Th2, and Th17 cells, each producing specific cytokines to support distinct immune functions. Th1 cells enhance cellular immunity, Th2 cells stimulate humoral immunity, whereas Th17 cells are involved in driving inflammatory responses and the progression of autoimmune diseases. Conversely, CD8+ cytotoxic T cells proliferate in response to infections and eliminate infected cells, thereby contributing to immunological memory (24). The pivotal role of T cells in the pathogenesis of NASH is unequivocally demonstrated by the absence of steatosis and hepatic inflammation in T cell-deficient mice (25, 26). In addition to CD4+ and CD8+ T cells, other T cell subsets, such as gamma delta (γδ) T cells, play a crucial role in the pathogenesis of NASH. Importantly, γδ T cells are found in peripheral tissues, including the liver, where they respond to inflammation and infections (27). The diverse T cell subsets significantly contribute to the inflammatory processes and disease progression in NASH, underscoring the complex and essential roles that T cells play in the development and progression of this condition.

### 3.1 CD4+ T helper cells

CD4+ T cells are increasingly recognized as a pivotal factor in the pathogenesis of NASH. Both preclinical and clinical research has demonstrated a marked accumulation of CD4+ T cells during NASH, suggesting that these cells are instrumental in promoting inflammation (28–32). Studies utilizing humanized mouse models have revealed that a high-fat, high-carbohydrate diet significantly elevates the number of CD4+ T cells, thereby contributing to disease progression. Notably, the depletion of CD4+ T cells through the use of therapeutic antibodies has been shown to reduce the production of inflammatory cytokines, disease activity, and fibrosis, underscoring the critical role that CD4+ T cells play in the clinical course of NASH (33). One of the primary mechanisms by which CD4+ T cells contribute to inflammation is through the secretion of proinflammatory cytokines. The migration and accumulation of CD4+ T cells in the liver are facilitated by the interaction between T cell integrin α4β7 and its ligand, mucosal vascular addressing cell adhesion molecule-1 (MAdCAM-1), which is expressed on liver endothelial cells and colonic mucosa. In mouse models, blocking this migration mechanism has been shown to effectively reduce CD4+ T cell accumulation and alleviate liver inflammation (34). Thus, the role of CD4+ T cells in the pathogenesis of NASH is intrinsically linked to their ability to promote the release of inflammatory cytokines and the accumulation of inflammatory cells within the liver tissue. Different subsets of CD4+ T cells appear to have distinct roles in the NASH pathophysiology. Specifically, Th1 and Th17 cells have been implicated in the promotion of inflammation, while Treg cells may exert a suppressive effect on the inflammatory process. Understanding the specific functions and contributions of these CD4+ T cell subsets is crucial for identifying novel therapeutic targets and developing effective strategies for the treatment of NASH.

#### 3.1.1 Th1 cells

Th1 cells, a subset of CD4+ T cells, are primarily characterized by their production of interferon (IFN)-γ, IL-2, and TNF-α. These cytokines play a crucial role in the activation of STAT4 and STAT1 in effector cells, leading to pro-inflammatory effects (24). Prior investigations have demonstrated an increase in hepatic Th1 cells in animal models of NASH (33, 35). In a study utilizing a steatohepatitis model induced by a methionine- and choline-deficient high-fat diet, mice deficient in IFN-γ exhibited reduced liver steatosis and fibrosis compared to wild-type mice (32). These findings suggest that Th1 cells play a significant role in the progression of NASH. Published data have consistently reported an increased proportion of Th1 cells in both peripheral blood and hepatic tissues of pediatric and adult patients with NASH compared to healthy control subjects (31). However, the differences in Th1 cell count between patients with NAFLD and those with NASH have not been found to be statistically significant (36). Despite this, the expression of genes encoding cytokines that promote the differentiation and activation of T cells towards a Th1 phenotype is markedly upregulated in NASH compared to NAFLD (37). Furthermore, the percentage of Th1 cells producing IFN-γ has been shown to exhibit a positive correlation with insulin resistance in obese patients, as evidenced by elevated levels of leptin, insulin, and homeostatic model assessment of insulin resistance (HOMA-IR) values (38). Additionally, the presence of Th1 cells in visceral adipose tissue has been found to be significantly correlated with plasma C-reactive protein (CRP) levels, suggesting their involvement in obesity-driven inflammatory processes (39).

IFN-γ plays a significant pathogenic role in the liver by inducing hepatocyte apoptosis and upregulating the expression of chemokines, such as C-C motif chemokine ligand 20 (CCL-20), and their receptors on hepatocytes (40, 41). Additionally, IFN-γ activates Kupffer cells, the liver-resident macrophages, further amplifying the inflammatory response (42). In addition, IFN-γ-induced chemokine C-X-C motif chemokine ligand 10 (CXCL10) is responsible for the recruitment of T cells expressing C-X-C motif chemokine receptor 3 (CXCR3). Notably, elevated serum levels of CXCL10 have been observed in patients with NASH. Studies in murine models have also demonstrated that deficiency in CXCR3 or deletion of CXCL10 attenuates liver inflammation, injury, and fibrosis (43, 44). In summary, Th1 cells, through their production of IFN-γ and other cytokines, substantially contribute to the inflammatory milieu in NASH.

#### 3.1.2 Th2 cells

Th2 cells, characterized by the expression of the transcription factor GATA3, primarily secrete IL-4, IL-5, and IL-13 through the activation of STAT5 and STAT6 signaling pathways. These cytokines exhibit dual roles in the context of liver disease. An increased number of Th2 cells has been reported in the peripheral blood of patients with NAFLD compared to healthy controls (36). However, no significant differences in Th2 cell count have been observed in either peripheral blood or liver tissue between patients with NASH, NAFLD, or healthy controls (31, 37). Th2 cells are typically associated with anti-inflammatory effects (45, 46); however, they can also contribute to the development of liver fibrosis, particularly through the actions of IL-13. Elevated hepatic expression of IL-13 and its receptor, IL-13RA2, have been detected in patients with NASH. Importantly, IL-13 signaling, especially via IL-13RA2 expressed by hepatic stellate cells (HSCs), has been implicated in the promotion of fibrosis (47). In a recent study, it has also been reported that IL-33 induces Th2 polarization and activates hepatic stellate cells in an IL-13-dependent manner, thereby promoting fibrosis (48). In summary, although Th2 cells exert a protective function through the modulation of inflammation, they also intricately participate in the pathogenesis of NASH by promoting liver fibrosis via IL-13 signaling pathways. The precise role of these cells in the development of NASH remains unclear, highlighting the need for further research to elucidate the balance between their anti-inflammatory and pro-fibrotic effects.

#### 3.1.3 Th17 cells

Th17 cells, a distinct subpopulation of CD4+ T helper cells, are characterized by their production of proinflammatory cytokines, including IL-17A, IL-22, and IL-23 (49), and their expression of the lineage-specific transcription factor retinoic acid receptor-related orphan receptor γt (RORγt) (50). Extensive research has implicated Th17 cells in the pathogenesis of various autoimmune and inflammatory disorders, with NASH being a notable example. In patients with NASH, the count of Th17 cells is significantly higher compared to healthy controls (36, 51, 52). A similar increase is also observed in the hepatic parenchyma and peripheral blood of animal models of NAFLD and NASH (53–55). Notably, the Th17 cell count decreases significantly 12 months after bariatric surgery (36), suggesting a potentially reversible association with disease severity. In the context of NAFLD, steatotic hepatocytes exhibit a strong response to IL-17A, leading to the upregulation of IL-17RA and increased production of proinflammatory and fibrogenic cytokines (e.g., IL-6 and TNF-α) (55–57). Additionally, IL-17A stimulates non-parenchymal liver cells to produce pro-inflammatory cytokines and chemokines, activating HSCs and promoting the expression of pro-fibrotic genes such as collagen type I alpha 1 and alpha-smooth muscle actin (58, 59). Multiple studies have demonstrated that blocking IL-17A or its receptor IL-17RA results in reduced hepatic steatosis, inflammation, and fibrosis, highlighting its significance in the pathogenesis of NASH (51, 54, 60).

Th17 cells also play a crucial role in the exacerbation of liver inflammation through the IL-17-dependent upregulation of the chemokine CXCL10, which contributes to the recruitment of macrophages (55). Inhibiting IL-17 signaling has been shown to reduce Kupffer cell activation and decrease the levels of pro-inflammatory cytokines, providing further evidence for the pro-inflammatory role of Th17 cells in the pathogenesis of NASH (60). A recent investigation employing single-cell RNA sequencing and multiparameter flow cytometry techniques conducted a comprehensive analysis of liver-infiltrating CD4+ T cells in patients with NAFLD and NASH. The findings revealed that the prevalence of Th17 cells was significantly higher in NASH patients presenting with fibrosis compared to those without fibrotic changes. Furthermore, the study demonstrated a positive correlation between the expression levels of IL-17A in the liver and the severity of fibrosis (61). In a recent study, a distinct population of CXCR3+Th17 cells within hepatic Th17 cells has been identified and termed inflammatory hepatic Th17 cells. These cells are suggested to play an active role in inflammation by producing IL-17A, IFN-gamma, and TNF-alpha (62). In summary, the increased Th17 cell count and their enhanced production of IL-17A are considered critical factors contributing to the progression and severity of NASH. These findings collectively suggest that targeted modulation of Th17 cell activity and function may represent a promising therapeutic strategy for the management of NASH and its associated fibrotic complications.

#### 3.1.4 Regulatory T cells

Regulatory T cells (Tregs), characterized by the expression of CD4+, CD25+, and FOXP3+ markers, play a pivotal role in maintaining immune tolerance and modulating immune homeostasis (63). These cells exert their immunosuppressive functions through the release of cytokines such as IL-10, TGF-β, and IL-35 (64). The differentiation of Tregs is dependent on the activation of signal transducer and activator of transcription 5 (STAT5) by IL-2 (65).

Studies in animal models have demonstrated a reduction in liver Treg cell populations in the context of NAFLD (66–68). In murine models of NASH, depletion of Tregs exacerbates disease severity, obesity, and insulin resistance (66, 69), while reconstitution of Tregs has been shown to attenuate liver inflammation (70). In patients with NAFLD, both circulating and liver-resident Treg numbers are significantly lower compared to healthy controls, with a more pronounced decrease observed in those with NASH (36).

Oxidative stress plays a pivotal role in the progression from NAFLD to NASH by inducing apoptosis in Tregs (66). Tregs are particularly vulnerable to the effects of oxidative insults, which can disrupt the delicate balance between Th17 cells and Tregs. An increased Th17/Treg ratio has been shown to correlate with the severity of liver damage, inflammation, and fibrosis, serving as a marker for the transition from NAFLD to NASH (66, 71). Furthermore, a separate study has shown that MIG/CXCL9 promotes the proliferation of Th17 cells, thereby disrupting the Th17/Treg cell balance and potentially exacerbating NASH (72).

Tregs exhibit antifibrotic effects primarily through the secretion of IL-10 (73). Their depletion exacerbates liver fibrosis, which is accompanied by significant alterations in IL-10 production (74). Conversely, Treg-derived TGF-β exerts profibrotic effects (75). Recent research has also identified a maladaptive role for amphiregulin (Areg)-producing Tregs, which are prevalent in the livers of both mice and humans with NASH. Areg produced by Tregs promotes fibrosis via epidermal growth factor receptor (EGFR) signaling in hepatic stellate cells. In turn, the deletion of Areg in Tregs has been shown to reduce fibrosis, suggesting that while Tregs generally confer protection, their production of Areg may lead to metabolic disruptions in chronic liver disease (76). In a recent study using a NASH mouse model, it has been shown that intrahepatic Treg cells are elevated, and adoptive transfer of Tregs has been demonstrated to increase steatosis (77). Collectively, these results suggest that Tregs play a multifaceted role in the pathogenesis of NAFLD and NASH. Oxidative stress and inflammation can induce apoptosis in this cell population, while its reconstitution has been shown to mitigate liver inflammation. In addition, an imbalance between Tregs and Th17 cells is associated with the severity of NASH. The dual antifibrotic and profibrotic effects of Tregs at different stages of NASH underscore their complex functions, highlighting a critical area for future research.

#### 3.1.5 T follicular helper cells

T follicular helper (Tfh) cells are a subset of CD4+ T cells characterized by the surface expression of CXCR5, PD-1, and ICOS, as well as the secretion of cytokines such as IL-21, IL-4, and IL-17 (78, 79). These cells play a pivotal role in B cell activation and differentiation, making them essential participants in germinal center reactions (80).

While studies directly linking Tfh cells to NAFLD are lacking, recent research underscores the involvement of B cells, which are supported by Tfh cells, in the pathogenesis of metabolic syndrome and NAFLD. Intrahepatic B cells may contribute to NAFLD by promoting the secretion of TNF-α, IL-6, and IgG2a, as well as by activating CD4+ intrahepatic T cells (81). In C57BL/6 mice fed a high-fat diet, B cells initially induce inflammation in mesenteric adipose tissue by promoting type I macrophage differentiation and producing TNF-α. Subsequently, B cells migrate to the liver, where they trigger hepatocyte inflammation (82).

In approximately 60% of patients with NASH, B cell and T cell infiltration in the liver has been observed, along with the presence of focal aggregates resembling ectopic lymphoid structures (10, 83, 84). The size and prevalence of these aggregates is positively associated with lobular inflammation and fibrosis scores (83). The interactions between B cells and NAFLD suggest a potential role for Tfh cells, which support B cell function, in the development and progression of NAFLD. In addition, Tfh cells play a pivotal role in the maintenance of gut homeostasis through their interactions with B cells within germinal centers, which are essential for supporting the production of IgA (85). IgA, in turn, contributes to the preservation of microbial balance within the gut by inhibiting the overgrowth of specific microbial species and fostering diversity (86). Dysfunction in intestinal Tfh cells has been reported to occur in NASH. This results in a decreased IgA production, which consequently disrupts the delicate balance of gut homeostasis. The resulting gut microbiome imbalance may serve as a trigger for liver inflammation and the progression of NASH (87). While the infiltration of T and B cells and the presence of ectopic lymphoid structures in the liver have been well-documented in NASH, the specific role of Tfh cells in the pathogenesis of NASH remains largely unexplored. To better understand the pathogenesis of NASH, it is essential to investigate the roles of B cell activation and differentiation in modulating the immune balance between the gut and liver, as well as their contributions to liver inflammation.

#### 3.1.6 T helper cell plasticity

The ability of T helper lymphocytes to transform into different types depending on environmental conditions is known as T cell plasticity. For instance, Treg lymphocytes that have been differentiated ex vivo can convert into Th17 lymphocytes under proinflammatory stimuli (88). Moreover, Th17 cells can express CXCR3 on their surface within an inflammatory milieu and exhibit characteristics of the Th1 phenotype by secreting both IL-17 and IFN-γ. These cells, commonly referred to as Th17.1, are implicated in tissue damage and inflammation processes (89). Data on the role of T cell plasticity in the pathogenesis of NASH remain limited. However, it is known that the liver microenvironment can influence the acquisition of distinct phenotypes by CD4+ T cell subsets. In one study, the accumulation of liver CXCR3+ Th17 cells and their association with liver inflammation were reported in both humans and various mouse models of NAFLD. These cells were found to secrete TNF-α, IFN-γ, and IL-17, and exhibited a higher inflammatory and metabolic gene expression profile compared to classical CXCR3- Th17 cells (62). A separate study evaluating the functional properties of liver-infiltrating CD4+ T cells in NAFLD revealed that the onset on NASH is characterized by a population of multi-cytokine-producing CD4+ T cells (61).

### 3.2 Cytotoxic CD8+ T cells

CD8+ T cells exert cytotoxic effects through the secretion of perforin, granzyme, and pro-inflammatory cytokines, including TNF-α and IFN-γ (90). Concerning the pathogenesis of NASH, studies have demonstrated an increase in the number of active cytotoxic CD8+ T cells within the liver, accompanied by elevated levels of inflammatory mediators. This heightened CD8+ T cell activity has been correlated with the progression of liver damage (91). Conversely, during the development of HCC, there is a significant reduction in CD8+ T cell infiltration and their ability to produce cytotoxic factors, which may contribute to tumor progression (92). However, the precise alterations in CD8+ T cell phenotypes throughout the transition from NASH to HCC have not been fully elucidated and warrant further investigation. Prior studies have elucidated the diverse functional potentials and state transitions of hepatic CD8+ T cells during the progression of NASH. These cells exhibit phenotypic diversity, with distinct subsets such as effector-memory ((CD44highCD62Llow) and activated subsets (CD25highCD69high) contributing to liver tissue damage in NASH (29). Furthermore, single-cell RNA sequencing analysis of liver tissue from NASH patients has identified an abundance of CXCR6+ PD1high CD8+ T cells. This subset is characterized by high cytotoxic gene expression and is associated with the severity of liver inflammation and fibrosis. Notably, the depletion of these cells in a NASH mouse model results in reduced liver damage (93).

Understanding the dynamics of CD8+ T cell states is crucial during the development of NASH-associated HCC. Further functional research is necessary to elucidate how various CD8+ T cell populations modulate the adaptive immune response and contribute to hepatic carcinogenesis.

### 3.3 γδ T cells

The pathogenesis of NASH is a multifactorial process involving various factors such as lipid accumulation, gut microbiome imbalance, and oxidative stress. However, the transition from simple steatosis to NASH is primarily characterized by the onset of inflammation (94). Consequently, understanding the events that trigger the initiation of inflammation is of significant clinical importance. While both CD4+ and CD8+ T cells have been implicated in the pathogenesis of NASH in murine models and human studies, and have been shown to contribute to liver fibrosis (36, 55, 93, 95), these cells appear to be more closely associated with post-inflammatory mechanisms rather than the early immune response during the development of inflammation in the liver. Conversely, current evidence suggests that tissue-resident immune cells may play a crucial role in perceiving early metabolic stress and initiating the inflammatory response in the liver (96).

γδ T cells, characterized by a T cell receptor (TCR) composed of γ and δ chains, are considered part of the innate immune system (97). They represent a relatively small fraction (1–5%) of circulating lymphocytes in peripheral blood (98). However, these cells are commonly found in tissues such as the skin, gut, lung, and liver, where they exhibit a tissue-resident immune cell phenotype (99). Notably, γδ T cells are capable of recognizing a variety of antigens independently of major histocompatibility complex (MHC) molecules, allowing them to be activated without the assistance of antigen-presenting cells. Upon activation, γδ T cells exhibit various functional responses, including cytotoxic activity against target cells and the production of IL-17 and IFN-γ. These activities in turn contribute to pathogen clearance, tissue inflammation, and homeostasis (100). Substantial evidence supports a significant role of hepatic γδ T cells in the pathogenesis of NASH. In NASH mouse models, a marked increase in the number of intrahepatic γδ T cells and their production of IL-17 has been observed (101, 102). Conversely, in the absence of γδ T cells, a notable reduction in liver damage, lobular inflammation, and hepatic leukocyte infiltration has been reported (101, 102). Despite these findings, the precise mechanisms by which γδ cells influence liver inflammation during the transition from simple steatosis to NASH remain incompletely understood.

A study investigating the molecular mechanisms underlying disease progression along the NAFLD spectrum revealed elevated hepatic expression levels of NKG2D ligands and IL-17A in the early stages of NASH development. Furthermore, the authors suggested that the presence of IL-17+RORγt+ γδ T cells in the blood of patients with NAFLD is positively associated with liver stiffness. These findings highlight the potential utility of measuring these specific γδ T cell subsets as a non-invasive marker for NASH and fibrosis. Subsequent investigations in a NASH murine model demonstrated that metabolic stress upregulated the expression of NKG2D ligands in hepatocytes, facilitating their interaction with NKG2D-expressing γδ T cells. This in turn stimulated the secretion of IL-17A by γδ T cells, which induced the expression of chemokines in hepatocytes. Consequently, this process contributed to the recruitment of pro-inflammatory myeloid cells (e.g., liver macrophages and neutrophils), thereby exacerbating hepatic inflammation and fibrosis. Moreover, NASH mouse models deficient in NKG2D expression exhibited a marked attenuation of liver inflammation and fibrosis (103). In summary, the progression from simple steatosis to NASH is mediated by a multifaceted interplay of various factors, with inflammation playing a central role. γδ T cells appear to be crucial in initiating the inflammatory response to early metabolic stress.

## 4 B cells in NASH

B lymphocytes, historically recognized for their role in antibody production and cytokine secretion, have been identified as pivotal regulators of inflammation in the context of NAFLD and NASH. These cells are classified into two distinct subsets: B1 and B2. Notably, B1 cells originate from the fetal liver, whereas B2 cells derive from bone marrow progenitors (104).

Studies have demonstrated that mice consuming a high-fat, high-carbohydrate diet experience an elevation in B2 cell populations and a concomitant decrease in B1b cells. In a mouse model of NASH, intrahepatic B cells, predominantly of the B2 subtype, infiltrate the liver tissue. These cells display a proinflammatory gene expression profile characterized by increased secretion of IL-6 and TNF-α, thereby contributing to hepatic inflammation (105). Furthermore, in a NASH mouse model, depletion of B2 cells has been shown to attenuate Th1 cell activation and reduce the hepatic expression of IFN-γ, resulting in a partial amelioration of the inflammatory response (83).

B cell-activating factor (BAFF) is a cytokine secreted by various immune cells, including dendritic cells, neutrophils, activated T lymphocytes, and epithelial cells. It plays a crucial role in promoting B cell maturation and survival through its interaction with several receptors, namely BCMA, BAFF-R, and TACI, which are predominantly expressed on B cells (106). A growing body of evidence suggests that elevated BAFF levels are associated with adiposity and insulin resistance in obese individuals (107, 108). Moreover, BAFF levels have been shown to be positively associated with the severity of fibrosis in patients with NASH (109). In a NASH mouse model, neutralization of BAFF resulted in a reduction of lobular inflammation (83).

The presence of autoantibodies in patients with NAFLD is a frequently observed phenomenon and has been linked to disease complications (110, 111); however, the precise nature of this association remains elusive. A recent systematic review and meta-analysis study revealed that serum antinuclear autoantibodies were detected in approximately 25% of patients with biopsy-proven NAFLD. Moreover, these autoantibodies were associated with a significant risk of fibrosis, particularly in Eastern populations (112). Research is also ongoing to elucidate the mechanisms by which autoantibodies produced by B cells contribute to liver damage. Some studies suggest a potential link between oxidative stress-induced antigens and the production of IgG, which may fuel autoimmune reactions and promote fibrosis progression in NASH (83). Furthermore, experiments conducted in mice fed a high-fat, high-fructose diet demonstrated the involvement of autoantibodies targeting protein disulfide isomerase A3 in liver damage (113).

## 5 Triggering adaptive immune response in NASH

**Figure 3:**
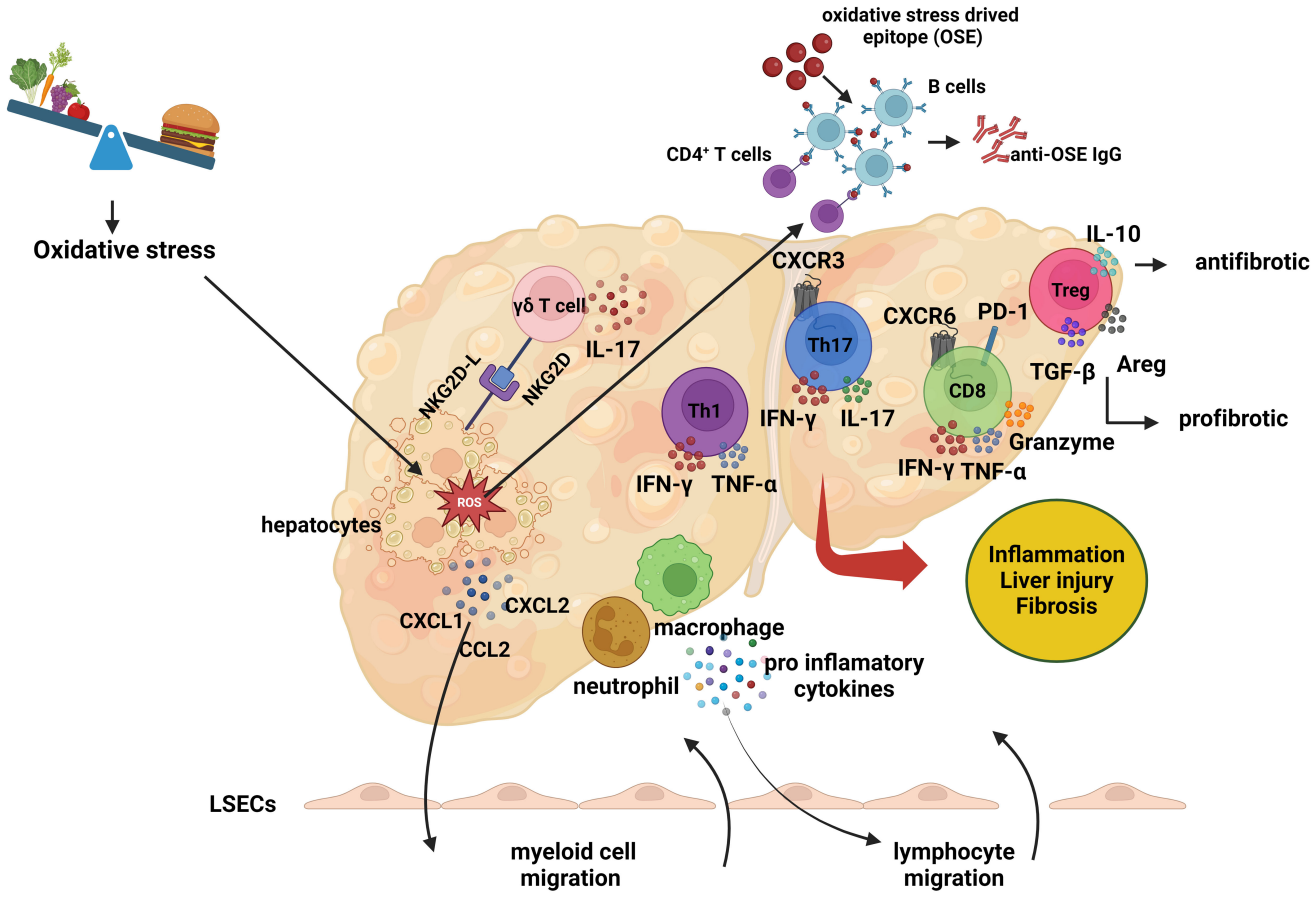
The role of adaptive immune response in the pathogenesis of non-alcoholic steatohepatitis. The role of adaptive immune response in the pathogenesis of non-alcoholic steatohepatitis. In the context of non-alcoholic steatohepatitis (NASH), lipid accumulation within hepatocytes leads to oxidative stress, which induces the expression of cell surface NKG2D ligands (NKG2D-L). This expression prompts gamma-delta (γδ) T cells to interact with the stressed hepatocytes and secrete interleukin-17 (IL-17). As a result of this interaction, hepatocytes produce chemokines, including C-X-C motif chemokine ligand 1 (CXCL1), C-X-C motif chemokine ligand 2 (CXCL2), and C-C motif chemokine ligand 2 (CCL2). These molecules trigger the migration of myeloid cells, such as macrophages and neutrophils, to the liver. The infiltrating myeloid cells secrete proinflammatory cytokines, which further promote the migration of lymphocytes. Additionally, Liver dendritic cells activate lymphocytes by presenting oxidative stress-induced epitopes (OSEs) to CD4+ T cells and B cells. Following this activation, helper T cells undergo Th1 or Th17 polarization, while B cells, upon presenting OSEs to CD4+ T cells, differentiate into plasma cells and synthesis anti-OSE IgG antibodies. Pro-inflammatory cytokines secreted by helper T and B cells also promote the activation of cytotoxic CD8+ T cells. These lymphocytes release cytokines such as interferon-gamma (IFN-γ), tumor necrosis factor-alpha (TNF-α), and IL-17, exacerbating the inflammatory environment and contributing to liver damage and fibrosis. The role of regulatory T (Treg) cells in the pathogenesis of NASH remains unclear. While Treg cells exhibit antifibrotic properties through the production of IL-10, they can also produce transforming growth factor-beta (TGF-β) and amphiregulin (AREG), which may promote fibrosis and liver damage. (Created with BioRender.com).

Elucidating the mechanisms underlying the adaptive immune response in the pathogenesis of NASH is of paramount importance. One crucial approach to achieving this goal is through the characterization of the antigenic stimuli that trigger lymphocyte responses. Oxidative stress and lipid peroxidation are prevalent features in the pathogenesis of NAFLD and NASH, serving as primary triggers for immune cell-mediated inflammation in the liver (**Figure 3**) (114). During the process of lipid peroxidation, oxidized phospholipids and reactive aldehydes not only function as damage-associated molecular patterns (DAMPs) that activate liver inflammation but also form antigenic structures known as oxidative stress-induced epitopes (OSEs) (115).

Oxidative stress also upregulates the expression of NKG2D ligands on hepatocytes, facilitating interactions with NKG2D-expressing γδ T cells and the subsequent secretion of IL-17. This interaction stimulates the secretion of chemokines by hepatocytes, which induces macrophage migration and activation in the liver (103). Activated macrophages release additional chemokines, attracting more monocytes to the region and leading to the secretion of pro-inflammatory mediators, promoting M1 macrophage polarization and resulting in oxidative damage (116, 117). Simultaneously, oxidative stress activates liver sinusoidal endothelial cells (LSECs), increasing the expression of adhesion molecules such as ICAM-1 and VCAM-1 (118). The chemokines induced by oxidative stress, in conjunction with the secretion of pro-inflammatory cytokines and the increased expression of adhesion molecules, promote the migration of leukocytes to the hepatic parenchyma.

OSEs formed as a result of oxidative stress are presented to lymphocytes by antigen-presenting cells, including macrophages and liver dendritic cells, thereby triggering lymphocyte activation (119). The activation signal, in turn, induces the expression of the costimulatory molecule OX40 on CD4+ T cells, leading to Th1 or Th17 polarization and the release of pro-inflammatory cytokines, which contribute to liver inflammation and fibrosis (10). The polarization and mechanisms of Treg cells in the liver remain unclear; while IL-10-secreting Treg cells have demonstrated anti-fibrotic effects, TGF-β or amphiregulin-secreting Treg cells exhibit pro-fibrotic properties, thereby promoting liver inflammation and fibrosis (120). Notably, B cells interact with CD4+ T cells by presenting OSEs, leading to their differentiation into plasma cells and the production of OSE-IgG (121). In addition, active CD4+ T cells induce the cytotoxic CD8+ T cell response, stimulating these cells to secrete pro-inflammatory cytokines (10). Collectively, these findings indicate that oxidative stress induces physiological changes in various liver cells, enhancing chemokine secretion and promoting leukocyte migration to the liver, thereby triggering inflammation.

## 6 Role of adaptive immune response in the transition from NASH to HCC

The mechanisms of adaptive immune response in the transition from NASH to HCC are complex and not fully elucidated. Current literature suggests that CD8+ T cells and T helper 17 (Th17) cells play a pathological role in this transition. Specifically, CD8+ T cells have been shown to play critical roles in NASH-HCC mouse models induced by long-term high-fat diet. It has been proposed that PD-1+CXCR6+ CD8+ T cells, which develop during the NASH stage, may promote HCC progression by killing hepatocytes in an auto-aggressive manner (93, 122). However, in the HCC stage, a decrease or exhaustion of functional activations of CD8+ T cells may contribute to hepatocyte resistance to tumor transformation and progression of HCC (91). The specific immune mechanisms that alter the frequency, activation, and functional properties of CD8+ T cells during the transition from NASH to HCC remain unclear and warrant further investigation.

Th17 cells also play a significant role in the development of NASH and HCC through IL-17 signaling. In mice fed a high-fat diet, DNA damage in hepatocytes leads to IL-17 release, causing neutrophil infiltration in adipose tissue, insulin resistance, steatohepatitis, and HCC (51). A recent study has demonstrated that IL-17A overexpression upregulates fibroblast activation protein-α expression, a critical regulator of HSC activation, thereby promoting liver fibrosis and tumorigenesis in both *in vivo* and *in vitro* experimental models (123). These findings indicate that IL-17A activates HSCs, fostering the development and progression of HCC, with enhanced expression of FAP and IL-17A further expediting this process (123). The study concluded that IL-17A promotes HCC by activating the STAT3 signaling pathway and increasing FAP expression in HSCs.

Considering the available data, it is evident that IL-17 plays a pathological role in various stages of NAFLD progression. However, the specific immune cells that modulate IL-17 and their functional activities during these transitions remain unclear. While Treg cells were initially thought to play a role, their different mechanisms of action in NASH development complicate this understanding. Although an imbalance between Treg and Th17 cells has been reported in the pathogenesis of NASH and HCC (68, 72), its functional mechanism is not fully elucidated. A comprehensive evaluation of T cell plasticity influenced by the inflammatory microenvironment in the liver can enhance our understanding of the pathological immune cell phenotypes across NAFLD stages.

In conclusion, the adaptive immune response plays complex roles in NASH-related hepatocarcinogenesis, with current research directing attention to the mechanisms of CD8+ T cells and Th17 cells. The application of novel high-dimensional sequencing tools to evaluate different subsets of immune cells that promote both NASH and HCC can significantly contribute to clarifying these complexities and illuminating effective mechanisms for targeted interventions.

## 7 Therapeutic options for modulating the immune response in NASH

Hepatocellular stress or injury in the liver activates immune cells, eliciting a robust inflammatory response. This process exacerbates damage to stressed hepatocytes, perpetuating inflammation. The dynamic nature of this inflammatory cascade and the intricate interactions between immune cells complicate our comprehensive understanding of NAFLD and NASH pathophysiology. Current pharmacological approaches targeting both the initiation and progression of hepatic inflammation, as well as modulation of complex immune cell interactions in the liver, are briefly examined.

Cenicriviroc (CVC), a CCR2/CCR5 antagonist, targets pro-inflammatory chemokine receptors critical in mediating inflammatory cell recruitment and liver inflammation. CVC inhibits monocyte infiltration, reducing inflammation and injury in NAFLD/NASH livers, and potentially impedes NASH-related fibrosis progression by suppressing pro-fibrotic cytokine production. Mouse models treated with CVC demonstrated inhibited macrophage accumulation and suppressed pro-fibrotic gene expression (124, 125). While a phase II clinical trial indicated positive long-term effects against fibrosis in advanced NASH patients (126), the latest phase III trial reported lack of efficacy in treating liver fibrosis, despite favorable safety and tolerability outcomes (127).

Lanifibranor, a pan-PPAR agonist, targets peroxisome proliferator-activated receptors (PPARα, PPARγ, PPARβ/δ) crucial in lipid and glucose metabolism regulation (128). PPAR activation promotes anti-inflammatory polarization of hepatic macrophages, offering a promising therapeutic approach (129, 130). In choline-deficient, amino acid-defined high-fat diet mice, lanifibranor reduced steatosis, inflammation, and fibrosis (130). A phase IIb clinical trial demonstrated lanifibranor’s efficacy in reducing Steatosis, Activity, and Fibrosis (SAF)-A scores in patients with NASH (131). Ongoing phase III trials (NATiV3 study; NCT04849728) evaluate lanifibranor in NASH patients with F2-F3 fibrosis. Elucidating its anti-inflammatory mechanisms could inform future therapeutic strategies.

Statins, potent inhibitors of 3-hydroxy-3-methyl glutaryl-CoA (HMG-CoA) reductase, exert multifaceted effects beyond cholesterol reduction. By binding to the enzyme’s active site, statins impede cholesterol synthesis, effectively lowering LDL cholesterol levels (132). However, their immunomodulatory properties extend to diverse aspects of adaptive immunity (133). Statins modulate T cell function through several mechanisms, including inhibition of lymphocyte function-associated antigen 1 (LFA-1), reducing T cell activation, proliferation, and migration (134), promotion of Th2 cell polarization while suppressing Th1 responses (135, 136), alteration of dendritic cell function and antigen presentation (137), enhancement of Treg function (138) and suppression of Th1 and Th17 cell differentiation (139). Statins demonstrate significant anti-inflammatory and antifibrotic effects in NAFLD/NASH pathogenesis. Simvastatin exhibits hepatoprotective properties by mitigating oxidative stress and inflammation, thereby ameliorating NAFLD (140). Atorvastatin suppresses the NLRP3 inflammasome pathway, reducing pro-inflammatory cytokines IL-1β and IL-18, and attenuates NASH progression by decreasing TNF-α levels (141). A 12-month atorvastatin regimen in NASH patients improved liver inflammation and steatosis (142), with long-term statin therapy showing liver condition improvements in most patients (143). Statins also display antifibrotic effects. For example, simvastatin improves NASH-related fibrosis prognosis by inhibiting hepatic stellate cell activation (144), whereas fluvastatin reduces hepatic fibrogenesis by modulating inflammation and oxidative stress *in vitro* and *in vivo* (145). In addition, clinical studies associate statin use with decreased risk of advanced liver fibrosis and NASH (146, 147). Despite substantial evidence of statins’ immunomodulatory effects, their impact on the pathogenesis of NASH remains incompletely understood. Further investigation into statin therapy’s influence on adaptive immune responses in patients with NASH and its correlation with clinical parameters could elucidate disease pathogenesis and inform novel therapeutic strategies.

Pentoxifylline (PTX) is a methylxanthine derivative with vasodilatory and anticoagulant properties. As a nonspecific phosphodiesterase inhibitor, PTX induces various cellular changes and suppresses inflammation by reducing TNF-α expression (148). It increases cyclic AMP (cAMP) signaling, which mediates hormonal and neurotransmitter effects and modulates cytokine-regulated signal transduction (149). PTX modulates both pro- and anti-inflammatory cytokines (150) and regulates the Treg/Th17 balance through cAMP signaling (151). PTX’s efficacy in NAFLD/NASH remains controversial. Some studies report positive effects in patients, including weight loss, improved liver function, and reduced inflammatory cytokines (148, 152). In NAFL mouse models, PTX decreased hepatic lipid accumulation, lowered blood lipids, and modulated M1 macrophage polarization to reduce inflammation (153). However, other clinical studies found no significant impact on NASH-associated metabolic markers (154, 155). While PTX demonstrates immunomodulatory effects, its precise molecular mechanisms in NASH treatment require further investigation. The conflicting results highlight the need for additional research to elucidate PTX’s therapeutic potential in NAFLD/NASH.

Glucagon-like peptide-1 (GLP-1), an incretin hormone produced by intestinal L cells, regulates postprandial glucose homeostasis and modulates insulin and glucagon secretion. GLP-1 receptor agonists (GLP-1 RAs) exert multifaceted effects, including glucose-dependent insulin secretion, glucagon suppression, and appetite reduction (156). Accumulating evidence demonstrates that GLP-1 RAs possess potent anti-inflammatory and immunomodulatory properties. These agents attenuate the production of pro-inflammatory cytokines (e.g., TNF-α, IL-1β, IL-6) while augmenting anti-inflammatory cytokine release (e.g., IL-10), effectively suppressing inflammatory cascades (157, 158). GLP-1 RAs also promote macrophage polarization towards an anti-inflammatory M2 phenotype (158, 159), inhibit lymphocyte migration, and mitigate inflammation (160). Recent investigations have revealed novel immunological mechanisms of GLP-1 RAs. They appear to function as negative costimulators in T cells (161) and enhance the immunosuppressive capabilities of Treg cells (162). Notably, these immunomodulatory effects occur independently of glycemic control, suggesting a broader spectrum of action beyond glucose regulation. Numerous studies indicate that GLP-1 RAs may significantly reduce weight, liver injury, and liver fat content (163, 164), and can slow the progression of fibrosis in patients with NASH (156, 165, 166). Despite not being approved for NAFLD treatment, recent studies have generated significant hope for their potential role in managing this condition. Further investigation into the immunomodulatory effects of GLP-1 RAs on NASH pathogenesis within the adaptive immune response framework could enhance confidence in this treatment approach. Additionally, ongoing clinical trials of weekly GLP-1 RAs in NAFLD will provide further clarity on the safety and efficacy of this treatment, potentially solidifying the position of GLP-1 RAs in the clinical management of NAFLD/NASH.

The complex pathophysiology of NAFLD and NASH, characterized by dynamic inflammatory processes and intricate immune cell interactions, necessitates pharmacological strategies that not only prevent hepatic inflammation and fibrosis but also modulate immune cell interactions. A deeper understanding of the molecular mechanisms of treatments with immunomodulatory potential will enhance their therapeutic efficacy and pave the way for innovative strategies in combating liver diseases.

## 8 Concluding remarks and future directions

Among the diverse array of immune cells implicated in the pathogenesis of NASH, T and B lymphocytes have emerged as pivotal players in orchestrating the inflammatory processes that drive disease progression. In this review, we have provided a comprehensive analysis of the intricate roles played by various T and B cell subsets, shedding light on their contributions to the development and progression of NASH. The distinct subpopulations of CD4+ T cells, namely Th1, Th17, and Treg cells, each exert specific effects on the inflammatory milieu characteristic of NASH. Th1 and Th17 cells, through their production of proinflammatory cytokines, play a pivotal role in promoting liver inflammation and fibrosis, underscoring their potential as therapeutic targets. Treg cells, renowned for their immunosuppressive functions, exhibit a complex role in NASH pathogenesis, with their dysregulation contributing to the exacerbation of liver inflammation and fibrosis. Tfh cells, although their direct involvement in NASH remains elusive, may influence disease progression by modulating B cell activation and differentiation. Furthermore, the plasticity of T helper cell subsets adds another layer of complexity to the pathogenesis of NASH, with dynamic transitions between phenotypes contributing to the perpetuation of inflammatory responses within the liver microenvironment. In parallel, cytotoxic CD8+ T cells and γδ T cells have emerged as significant contributors to NASH-associated inflammation. While CD8+ T cells exert direct cytotoxic effects on liver cells, γδ T cells initiate inflammation in response to early metabolic stress, thereby fueling disease progression. Moreover, B cells, traditionally recognized for their role in antibody production, have been shown to exhibit proinflammatory characteristics in the context of NASH. Notably, the activation of B cells and their secretion of cytokines have been implicated in the exacerbation of liver inflammation and fibrosis.

Therapies developed for NAFLD/NASH have primarily focused on modulating metabolic imbalances, oxidative stress, and innate immune responses. The potential effects of these treatments on adaptive immune cells have been discussed under the title ‘Therapeutic Options for Modulating the Immune Response in NASH’. However, developing treatment strategies that directly target adaptive immune responses could be a promising option in the future. Significant progress has been made in recent years regarding the role of T and B cells in the pathogenesis of NASH; however, their exact mechanisms of action remain unclear. Treatment strategies targeting the adaptive immune response are currently quite limited, and more functional research is needed to fully realize their potential.

In the realm of therapeutic strategies aimed at modulating the adaptive immune response, research centered on adhesion molecules facilitating lymphocyte migration, IL-17 production, which plays a pivotal role in liver inflammation and fibrosis, and pro-inflammatory B cells holds considerable promise for future advancements. Notably, a study demonstrated that neutralizing antibodies against the VAP-1 adhesion molecule, which promotes lymphocyte infiltration into the liver in NASH, effectively reduced the severity of steatohepatitis and delayed the onset of fibrosis in a NASH animal model (28). However, it has been suggested that this effect could inadvertently increase cytotoxicity and oxidative stress by inhibiting VAP1’s amine oxidase activity, thereby augmenting the production of aldehydes and hydrogen peroxide (167). Furthermore, research has shown that an anti-α4β7 monoclonal antibody significantly reduced liver inflammation and fibrosis by preventing the migration of α4β7-expressing CD4 T cells to the liver via binding to MAdCAM-1 in the liver and intestines (34).

One therapeutic strategy targeting the adaptive immune response in NASH involves the inhibition of IL-17 production, which is closely linked to inflammation and fibrosis. Experimental NASH animal models have consistently demonstrated that anti-IL-17 antibodies effectively reduce inflammation, liver damage, and disease progression (60, 168–170). Monoclonal antibodies targeting IL-17, such as secukinumab and ixekizumab, are commonly used in the treatment of rheumatic diseases and are being evaluated for their potential in strategies targeting IL-17 in psoriasis associated with NASH. Research suggests that biological agents inhibiting IL-17 can reduce the NAFLD fibrosis score and improve liver fibrosis by regulating hepatic inflammation (171, 172). A phase III clinical trial is ongoing to investigate the anti-inflammatory, anti-steatotic, and anti-fibrotic effects of secukinumab in non-alcoholic fatty liver disease coexisting with psoriasis (NCT042371169). Additionally, it has been proposed that secukinumab could enhance the autophagic cell death of HCC by reducing IL-17-induced BCL2 expression, thereby inhibiting HCC carcinogenesis (173). Collectively, these data indicate that IL-17 plays a crucial role in most stages of NAFLD progression and represents a potentially important target for therapeutic strategies.

Considering the pro-inflammatory properties of B cells in NASH pathogenesis and the association between BAFF levels and the severity of steatohepatitis and fibrosis, targeting B cells may prove to be an effective strategy in reducing liver inflammation and fibrosis. Treatments such as rituximab (anti-CD20) and belimumab (BAFF signaling inhibitor), which target B cells, have been successfully used in the treatment of various autoimmune diseases for years (174, 175). However, while B cell depletion therapies hold potential for improving NASH, they may also cause serious infections due to immunosuppression and toxicity, posing significant risks. Consequently, targeting BAFF might offer a safer and more effective therapeutic approach. In addition, it has been reported that the BAFF-neutralizing monoclonal antibody Sandy-2 inhibits hepatic B2 cell responses and improves established NASH in a mouse model (83). Additionally, BAFF depletion has been shown to reduce liver fat accumulation and improve insulin sensitivity in NAFLD mouse models (176). The interaction mechanisms between T and B cells in the development of NASH are not yet fully understood. Further functional studies are required to identify costimulatory molecules that play a role in this interaction, potentially leading to more selective therapeutic options and providing new and more effective approaches for the treatment of NASH.

In conclusion, the diverse and complex roles played by T cells and B cells in the pathogenesis of NASH highlight their critical importance in the development and progression of this complex liver disorder. Elucidating the intricate interplay between various T cell subsets, as well as their interactions with other immune cell populations, provides a promising avenue for the development of targeted therapeutic interventions in the management of NASH. By gaining a deeper understanding of the molecular mechanisms that drive T cell-mediated inflammation, researchers may uncover novel therapeutic targets, potentially ushering in a new era of more effective and personalized treatment strategies for this complex liver disorder.

## Author contributions

MC: Conceptualization, Visualization, Writing – original draft, Writing – review & editing. YY: Conceptualization, Supervision, Writing – original draft, Writing – review & editing.
